# Gut Microbiota Changes in Patients With Major Depressive Disorder Treated With Vortioxetine

**DOI:** 10.3389/fpsyt.2021.641491

**Published:** 2021-05-07

**Authors:** Xiaolin Ye, Dong Wang, Huaqian Zhu, Dahai Wang, Jing Li, Yanqing Tang, Jie Wu

**Affiliations:** ^1^Department of Gastroenterology, National Center for Children's Health, Beijing Children's Hospital, Capital Medical University, Beijing, China; ^2^Department of Psychiatry, The First Affiliated Hospital of China Medical University, Shenyang, China; ^3^Department of Pediatrics, China Medical University Affiliated With Shengjing Hospital, Shenyang, China

**Keywords:** major depression disorder, vortioxetine hydrobromide, gut microbiota, treatment, gut-brain axis

## Abstract

Vortioxetine hydrobromide is a common clinical medication for major depressive disorder (MDD). However, it remains unclear whether vortioxetine hydrobromide acts by affecting the structure and composition of gut microbiota. Here, we analyzed fecal samples from 28 healthy controls (HCs) and 26 patients with MDD before treatment with vortioxetine hydrobromide, at 4 weeks after treatment, and at 8 weeks after treatment. High-throughput pyrosequencing showed that, according to the Chao1 and Shannon indices, fecal bacterial α-diversity was higher in the patients with MDD than in the HCs (*p* < 0.05), but no significant differences were observed after vortioxetine hydrobromide treatment (*p* > 0.05). PCoA results revealed that the gut microbiota composition was significantly different between the MDD groups and HCs. Proteobacteria and Actinobacteria were strongly increased, whereas Firmicutes were significantly reduced in the MDD group compared with the HCs. After treatment with vortioxetine hydrobromide, Firmicutes were significantly increased, and the proportion of Bacteroidetes decreased. Most notably, *Lachnospira, Roseburia*, and *Faecalibacterium* were negatively correlated with the severity of depressive symptoms. Taken together, our data indicate changes in the fecal microbiota composition in MDD patients compared with HCs, and vortioxetine hydrobromide may treat MDD through regulation of the gut microflora.

## Introduction

Major depressive disorder (MDD) is a mental illness characterized by depression, decreased interest, cognitive impairment, and vegetative symptoms (such as sleep or appetite disorder) (1). As a common disease with high morbidity, MDD affects the health status of patients and significantly reduces quality of life. Moreover, MDD has become a serious social and medical problem because it causes a tremendous burden on families and society (2). Epidemiological studies have shown that MDD affects ~350 million people worldwide (3). In China, the prevalence of depression is ~6.87%, amounting to 90 million residents (4). It is estimated that by 2030, MDD will become one of the major causes of disease burden worldwide (5).

The pathogenesis of MDD is very complex, involving genetic, biochemical, neuroendocrine, immune and psychosocial environments, and other factors, and the monoamine hypothesis is the most common hypothesis used to explain MDD. It is believed that depression is mainly caused by abnormal monoamine transmitters between synapses, whereby insufficient concentrations of monoamine neurotransmitters, such as serotonin, norepinephrine, and dopamine, in the synaptic clefts lead to decreased neuronal activity, ultimately resulting in a depressive state. However, clinical antidepressant treatment based on the intracerebral monoamine neurotransmitter imbalance theory can only relieve symptoms in 35% of patients. Thus, although intracerebral factors are important for the pathogenesis of depression, there is an urgent need to determine the factors associated with the external environment and elucidate their mechanisms of action.

In recent years, it has become increasingly evident that MDD is closely related to the gut microbiota and that fecal microbiota transplantation significantly improves depression (6). A two-way communication pathway between the brain and gut has long been recognized, known as the microbiota-gut-brain axis, and it occurs through a variety of pathways, including the immune system, neuroendocrine system, the vagus nerve and the enteric nervous system, and microbial metabolites, such as short-chain fatty acids, peptidoglycan, and branched-chain amino acids (7). Changes in the species, abundance, and quantity of gut microflora in MDD patients cause an imbalance of microflora, leading to the production of inflammatory factors. A reduction in the 5-hydroxytryptamine (5-HT) level in tryptophan (TRP) metabolism results in neuronal dysfunction and the generation of toxic metabolites downstream of neurons, causing neuronal dysfunction in specific parts of the central nervous system (8, 9). A recent report on the use of probiotics in MDD patients showed improvement in outcome measures of depression or anxiety after probiotics intervention (10). These results suggest that MDD may influence the composition of the gut microbiota and that targeting the gut microbiota has the potential to treat MDD.

Vortioxetine hydrobromide is a new antidepressant that has been widely used in clinical practice (11, 12). The results of existing clinical studies have shown that vortioxetine hydrobromide helps improve the affective symptoms of depression and improves the cognitive symptoms of patients with depression; this improvement is independent of improvements in affective symptoms. This drug has been shown to facilitate comprehensive functional recovery and provides more choices and hopes for patients with depression (13–15). According to the pharmacodynamic data and preclinical studies, it is thought to exert therapeutic effects through the combination of inhibiting the serotonin transporter (SERT) and modulating 5-HT receptor activity (12). Based on the important role of the gut microbiota in the pathogenesis of MDD, we hypothesize that during the treatment of MDD with vortioxetine hydrobromide, changes in gut microflora are induced that improve the course of MDD. The purpose of this study was to comprehensively understand the characteristics of gut microbiota in patients with MDD and further explore the mechanisms of the occurrence and development of MDD. We further sought to analyze changes in the gut microbiota as a result of vortioxetine hydrobromide in the course of MDD treatment and to provide a new theoretical basis for the clinical treatment of MDD with vortioxetine hydrobromide.

## Materials and Methods

### Subject Selection

**Figure 1:**
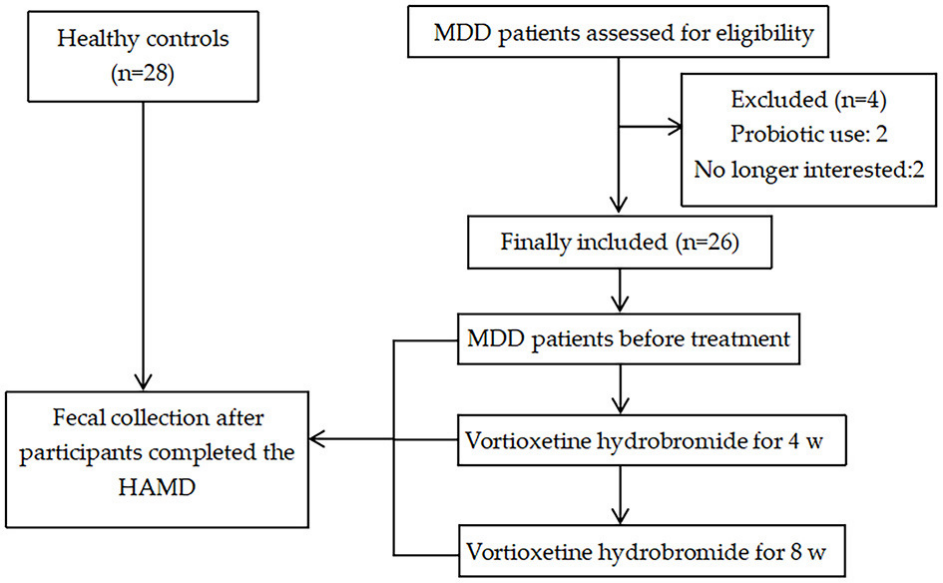
Flowchart for participant recruitment and sample collection process.

The protocol for this study was approved by the Ethics Committee of China Medical University (Approval No. 2020-24-2). After receiving a written description of the aims of this study, all participants provided written informed consent prior to enrollment. The basic information of the participants was registered, fecal samples were collected, and the numbers were maintained. Next, patients with MDD were treated with vortioxetine hydrobromide (dosage of 10 mg once daily and taken with food or on an empty stomach). Stool specimens were collected at 4 and 8 weeks after treatment and numbered for preservation after treatment. The recruitment of participants and the sample collection process are depicted in Figure 1.

Fifty-four patients (age, 18–50 years) were recruited from the First Affiliated Hospital of China Medical University in Shenyang, Liaoning from October 2019 to January 2020 (Table 1). The study was approved by the Ethics Committee of the First Affiliated Hospital of China Medical University. Subjects who participated in this study were informed of the aims of the study and provided with related information. Written informed consent was obtained for each participant.

**Table 1:** Patient characteristics.

**Variable**	**Healthy controls (*n* = 28)**	**MDD patients (*n* = 26)**	***P*-value**
Female, no. (%)	21 (75%)	21 (81%)	0.61^b^
Age (years; mean ± SD)	26.04 ± 7.83	26.04 ± 7.83	0.14^a^
BMI (mean ± SD)	21.59 ± 3.48	19.78 ± 2.12	<0.05^a^
HAMD (0 w)	1.29 ± 1.3	27.92 ± 2.77	<0.01^a^
HAMD (4 w)	–	16.77 ± 4.01	
HAMD (8 w)	–	9.23 ± 3.27	

a *Student's t-test*.

b *chi-square test*.

*MDD, major depressive disorder; SD, standard deviation; BMI, body mass index; HAMD, Hamilton's Depression Scale*.

The inclusion criteria for the healthy controls (HCs) were as follows: (1) no DSM-IV axis I or axis II disorders; (2) no family history of mental illness; (3) no use of any psychiatric drugs; (4) age 18–50 years (no sex requirement); (5) no blood relationship with the patients; and (6) matched based on sex, age, education, and met the requirements to read and understand the written word and answer the Hamilton Questionnaire.

The inclusion criteria for patients with MDD were as follows: (1) after passing a consistency test administered by two psychiatrists with extensive clinical experience and titles exceeding associate chief physician, patients aged 18 years and older were interviewed by structured clinical interviews to determine the corresponding diagnosis according to the Diagnostic and Statistical Manual of Mental Disorders-IV (DSM-IV) axis I criteria; (2) age between 18 and 50 years; (3) elementary school education or higher; (4) Han nationality (no sex requirement); (5) Hamilton Depression Scale (HAMD-17) score ≥ 24; and (6) 18.5 < BMI < 24.

The exclusion criteria were as follows: (1) major physical disease history, especially diseases that may be associated with brain tissue changes, such as hypertension, diabetes or metastatic tumors, liver cirrhosis, fatty liver, irritable bowel syndrome (IBS), and inflammatory bowel disease; (2) unstable physical illness, severe asthma, abnormal nervous system history, including major head trauma (continuous loss of consciousness lasting longer than 5 min), epilepsy, cerebrovascular disease, brain tumors and neurodegenerative diseases; physical diseases that may cause mood disorders, such as multiple sclerosis and thyroid diseases, and autism or extensive developmental disorders; (3) use of antidepressant or antipsychotic drugs during the previous 2 weeks; (4) long-acting antipsychotic drugs or electroshock therapy (MECT) within the previous month; (5) pregnant and lactating women; (6) stress events in the previous week; (7) drug abuse or alcohol abuse in the previous year; use of antibiotics, probiotics, or synbiotics in the previous month; and known active bacterial, fungal or viral infections; and 8) HAMD-17 score did not decrease by 50% after 4 weeks of vortioxetine hydrobromide treatment in MDD patients.

### Depressive Symptoms Measures

To assess depression, two psychiatrists with extensive clinical experience administered the HAMD-17. Some items in the HAMD-17 adopt a 5-point scoring system, i.e., 0–4, and other items adopt a 3-point scoring system, i.e., 0–2. The higher the score for each item, the more obvious the symptom, and the higher the total score, the more obvious the depressive symptoms. After reviewing the collected questionnaires for issues, the responses from qualified questionnaires were entered into Excel tables by members of the study team. The HAMD was administered on the day of the visit.

### Fecal Sample Collection and DNA Extraction

After the HAMD score was completed, fresh stool samples of the study subjects were collected with disposable feces collectors and frozen in an ultra-low temperature freezer at −80°C. Total bacterial DNA was extracted from samples using a Power Soil DNA Isolation Kit (MO BIO Laboratories, Carlsbad, CA, USA) following the manufacturer's protocol as previously described (16). DNA quality and quantity were assessed using the 260/280 nm ratio and the 260/230 nm ratio, respectively. Then, DNA was stored at −80°C until further processing.

### Polymerase Chain Reaction and Illumina High-Throughput Sequencing

The V3-V4 region of the bacterial 16S rRNA gene was amplified with a common primer pair (Forward primer, 5′-ACTCCTACGGGAGGCAGCA-3′; reverse primer, 5′-GGACTACHVGGGTWTCTAAT-3′) combined with adapter sequences and barcode sequences. PCR amplification was performed as described previously (17) in a total volume of 50 μl; each reaction contained 10 μl of buffer, 0.2 μl of Q5 High-Fidelity DNA Polymerase, 10 μl of High GC Enhancer, 1 μl of dNTPs, 10 μM each primer, and 60 ng of genomic DNA. The thermal cycling conditions were as follows: initial denaturation at 95°C for 5 min, followed by 15 cycles at 95°C for 1 min, 50°C for 1 min, and 72°C for 1 min, with a final extension at 72°C for 7 min. The PCR products from the first-round PCR were purified using VAHTSTM DNA Clean Beads. Second-round PCR was then performed (total reaction volume, 40 μl); each reaction contained 20 μl of 2× Phusion HF MM, 8 μl of ddH_2_O, 10 μM each primer, and 10 μl of PCR products from the first-round PCR. The thermal cycling conditions were as follows: initial denaturation at 98°C for 30 s, followed by 10 cycles at 98°C for 10 s, 65°C for 30 s min, and 72°C for 30 s, with a final extension at 72°C for 5 min. All PCR products were quantified using Quant-iT™ dsDNA HS Reagent and then pooled. High-throughput sequencing analysis of bacterial rRNA genes in the purified pooled sample was performed using an Illumina HiSeq 2500 platform (2 × 250 paired ends) at Biomarker Technologies Corporation, Beijing, China (18).

### Bioinformatics and Statistical Analysis

Using FLASH v1.2.7 software, the reads for each sample were spliced by overlapping, and the resulting spliced sequences were the raw tags. Using Trimmomatic v0.33 software, the raw tags obtained by splicing were filtered to obtain high quality clean tags. UCHIME v4.2 software was used to identify and remove chimeric sequences and obtain effective tags. Operational taxonomic unit (OTU) clustering was performed based on 97% similarity using UPARSE, and each representative sequence was used for annotation using RDP Classifier. Alpha diversity analysis was used to study species diversity within individual samples. The Chao1 and Shannon indices for each sample at the 97% similarity level were statistically analyzed, and rarefaction curves were plotted. Beta diversity analysis was used to compare species diversity (community composition and structure) differences among different samples. Principal coordinate analysis (PCoA) plots at corresponding distances were obtained based on a distance matrix, i.e., unweighted UniFrac distance metrics. Linear discriminant analysis effect size (LEfSe) analysis was carried out for comparisons among intergroup samples. An alpha significance level of 0.05 and an effect-size threshold of 3.5 were used for all biomarkers. Statistical analyses were performed using SPSS ver. 21.0 data analysis software (SPSS Inc., Chicago, IL, USA). All tests for significance were two sided, and *p* < 0.05 indicated a significant difference.

## Results

### Overall Structure of the Fecal Bacterial Communities

**Figure 2:**
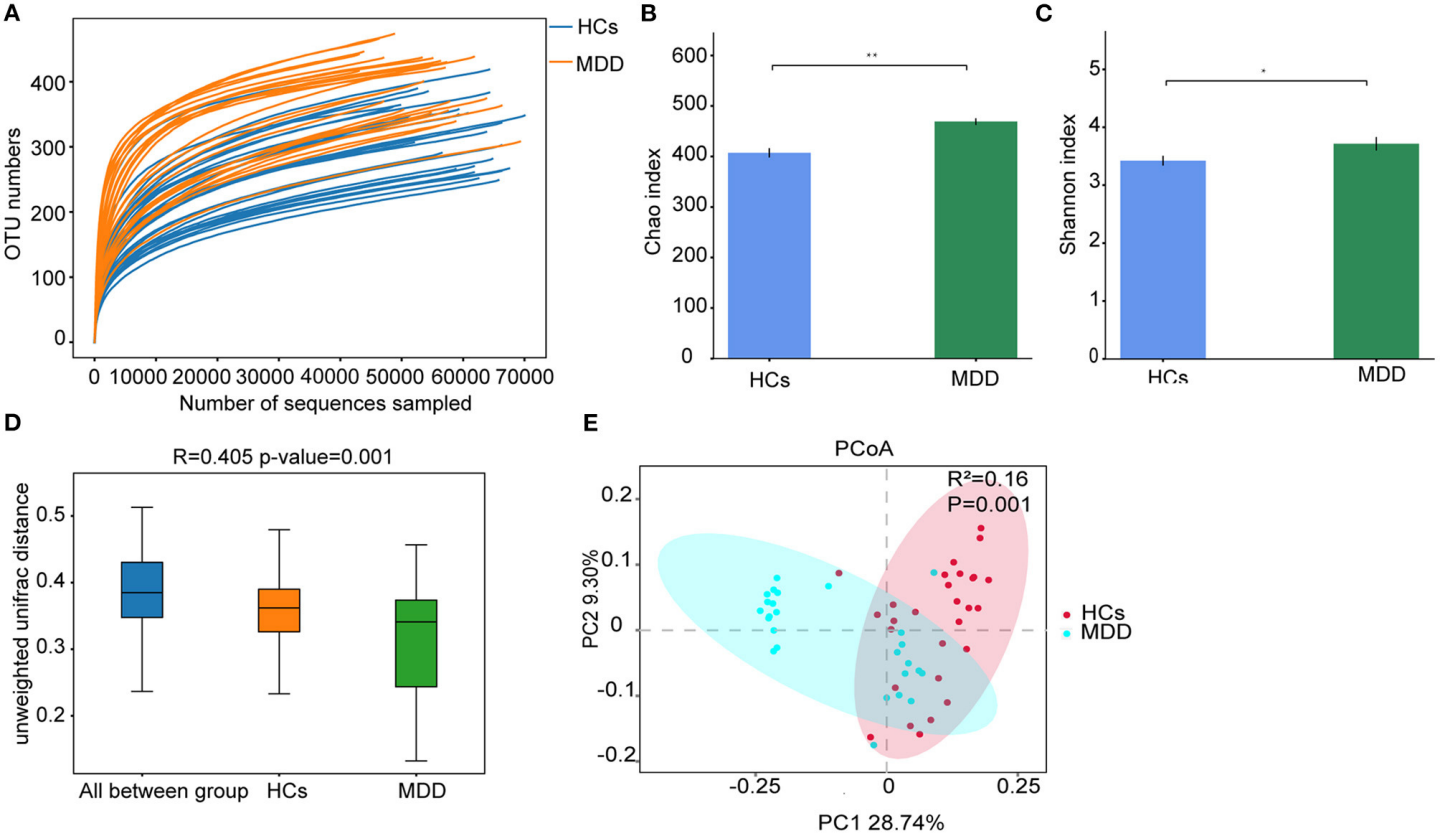
MDD leads to significant structural and functional changes in the gut microbiota. **(A)** Rarefaction curve from MDD and HCs. **(B)** α-Diversity on the Chao index between groups. **(C)** α-Diversity on the Shannon index between groups. **(D)** β-Diversity on unweighted UniFrac between groups. **(E)** PCoA of the gut microbiota metagenomes between groups. *p < 0.05, **p < 0.01.

Rarefaction curves indicated that the sequencing depth was sufficient to capture all bacterial species and for downstream analysis (Figure 2A). Richness estimates were obtained from the observed number of species by extrapolation using Chao1 and Shannon indices. The results showed that bacterial diversity was significantly higher in the MDD group than in the HCs (*p* < 0.05) (Figures 2B,C). Furthermore, β-diversity calculated with the Unweighted UniFrac (*p* = −0.001) algorithms indicated that the MDD and HC groups had significant structural differences by the first dimension of space distance (Figure 2D). PCoA results revealed that the first two principal factor analyses explained 28.74 and 9.30% of the total variability (*p* < 0.05). As shown in Figure 2E, at the OTU-based single sample level, the gut microbiota composition in the MDD and HC groups could be discriminated by PCoA.

### Altered Microbiota Composition in MDD Patients

**Figure 3:**
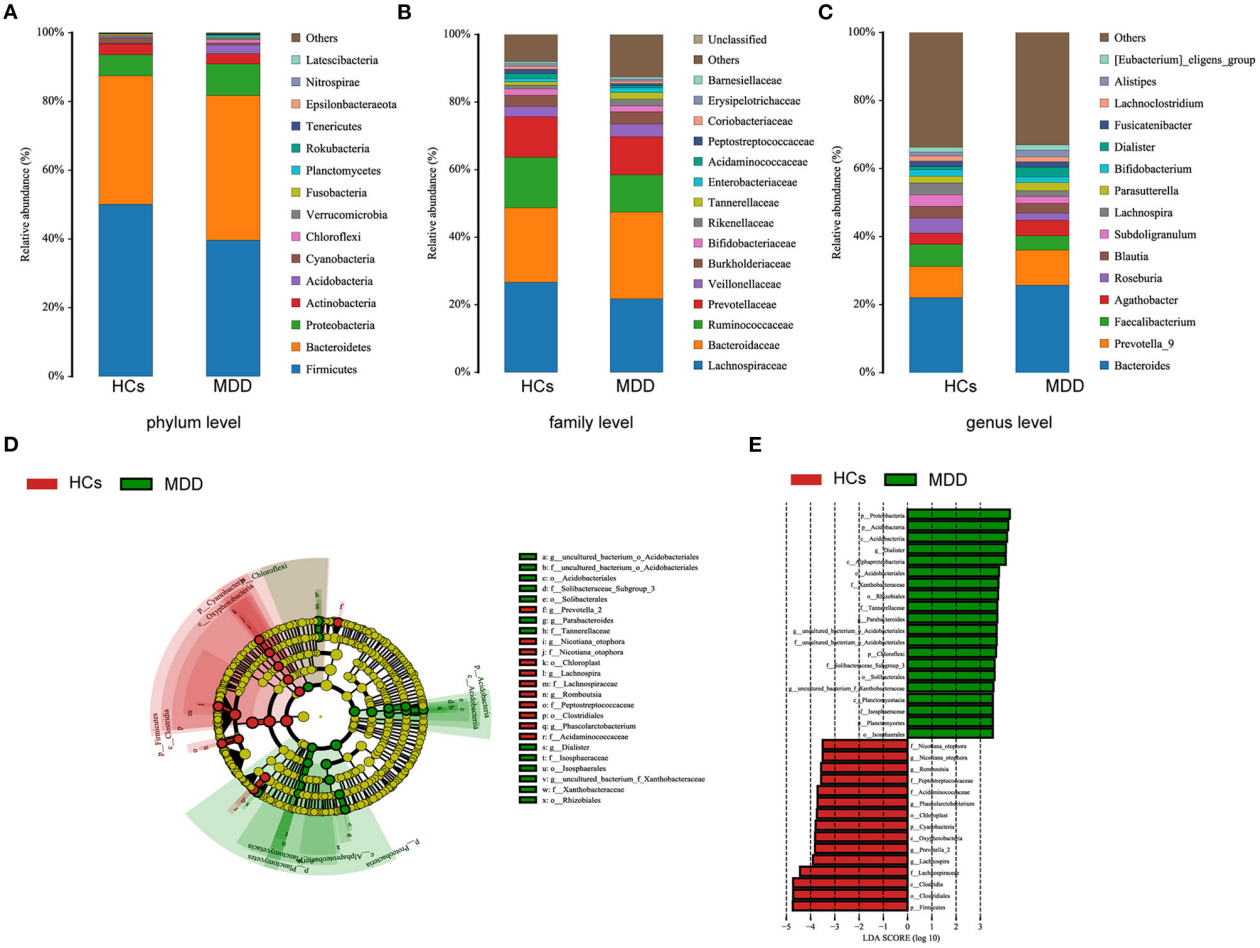
Taxonomic differences in fecal microbiota between HCs and MDD patients. Comparison of relative abundance at the bacterial phylum **(A)**, family **(B)** and genus **(C)** levels between HCs and MDD patients. LEfSe identified the most differentially abundant taxa between HCs and MDD patients. The brightness of each dot is proportional to its effect size **(D)**. Taxa enriched in HCs are indicated with a negative logarithmic discriminant analysis (LDA) score (red), and taxa enriched in MDD patients have a positive score (green). Only taxa meeting an LDA significance threshold > 3.5 are shown **(E)**.

The MDD patients and HCs exhibited statistically significant differences with regard to the four dominant phyla: Firmicutes, Bacteroidetes, Proteobacteria, and Acidobacteria (Figure 3A). There were 14 statistically significant differences between the MDD patients and HCs at the family level. The relative proportions of Bacteroidaceae, Veillonellaceae, Burkholderiaceae, Rikenellaceae, Enterobacteriaceae, Barnesiellaceae, and Tannerellaceae were significantly higher in the MDD patients than in the HCs; we also found significantly lower levels of Peptostreptococcaceae, Acidaminococcaceae, Lachnospiraceae, Prevotellaceae, Ruminococcaceae, Erysipelotrichaceae, and Bifidobacteriaceae in the MDD patients than in the HCs (Figure 3B). At the genus level, the abundances of *Bacteroides, Alistipes*, and *Prevotella-9* were higher and those of *Faecalibacterium, Roseburia*, and *Bifidobacterium* were lower in MDD patients than HCs (Figure 3C).

The metagenome analysis LEfSe approach was applied to identify the key phylotypes responsible for the differences between the MDD patients and HCs. At the phylum level, Proteobacteria and Acidobacteria were more abundant in the MDD patients, and Firmicutes were more abundant in the HCs. At the genus level, *Dialister, Parabacteroides, Aquisphaera*, and *Bacillus* were most abundant in the MDD, while *Romboutsia, Phascolarctobacterium, Roseburia, Prevotella-2*, and *Lachnospira* were most abundant in the HC, contributing to the differences in intestinal microbiota between the MDD patients and HCs (Figures 3D,E).

### Vortioxetine Hydrobromide Treatment Leads to Significant Structural Changes in the Gut Microbiota

**Figure 4:**
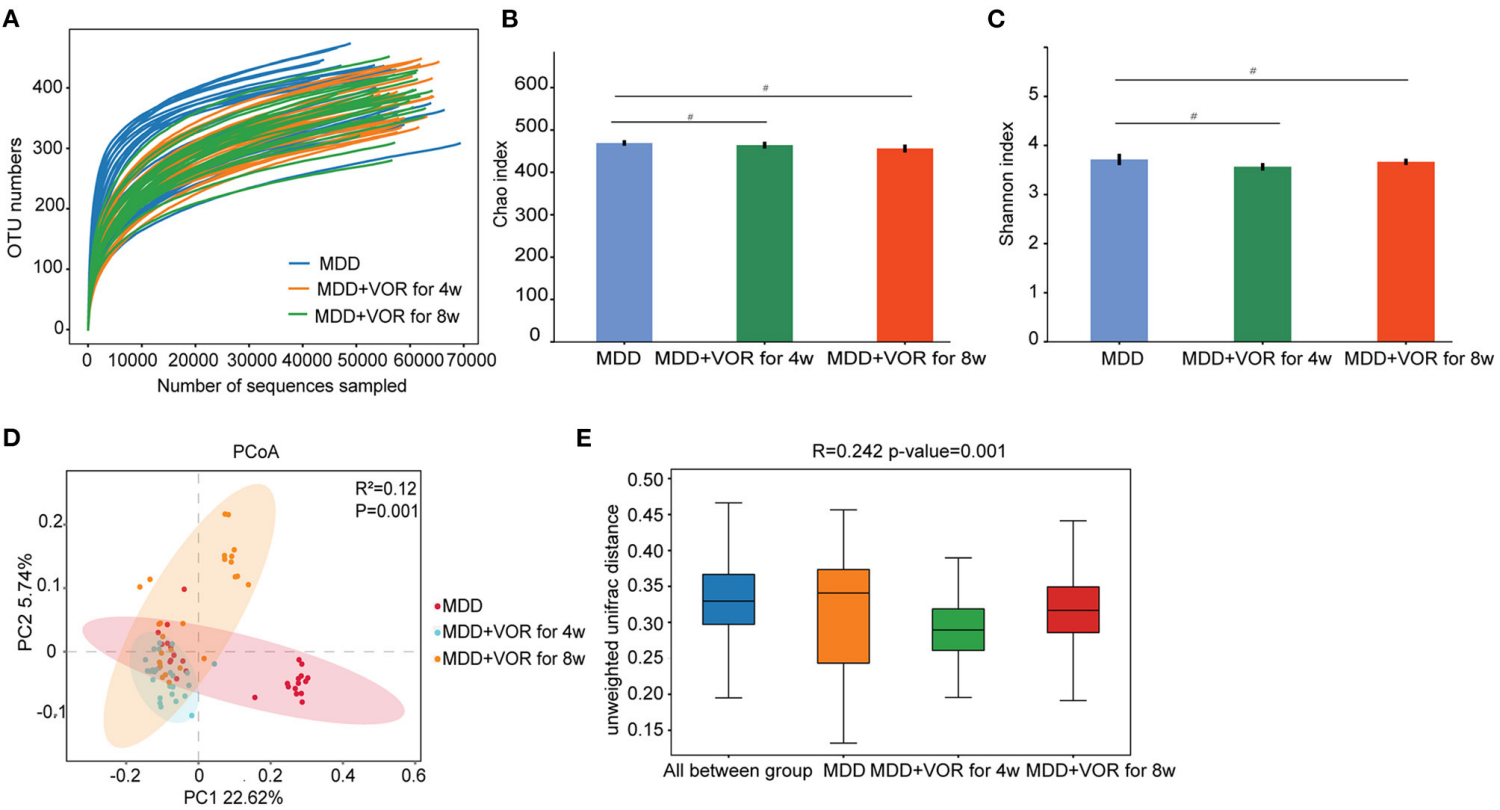
Alpha and beta diversity measures in MDD patients before and after vortioxetine hydrobromide treatment. **(A)** Rarefaction curve between groups. **(B)** α-Diversity on the Chao index between groups. **(C)** α-Diversity on the Shannon index between groups. **(D)** PCoA of the gut microbiota metagenomes between groups. **(E)** β-Diversity on unweighted UniFrac between groups. #*p* < 0.05.

The rarefaction curve was relatively flat, suggesting a relatively sufficient amount of sequencing for each sample (Figure 4A). The Chao1 index and Shannon index showed no significant differences in species richness or species evenness among the three groups of samples (*p* > 0.05) (Figures 4B,C). The PCoA results showed that the first two principal components explained 22.62 and 5.74% of the total variability (Figure 4D). β-Diversity calculated with the Unweighted UniFrac algorithms indicated that the three groups had significant structural differences (*p* < 0.01) (Figure 4E). The above results indicate that there were significant differences in microbial community diversity before and after vortioxetine hydrobromide treatment.

### Composition of the Gut Microbiota in MDD Patients Before and After Vortioxetine Hydrobromide Treatment

**Figure 5:**
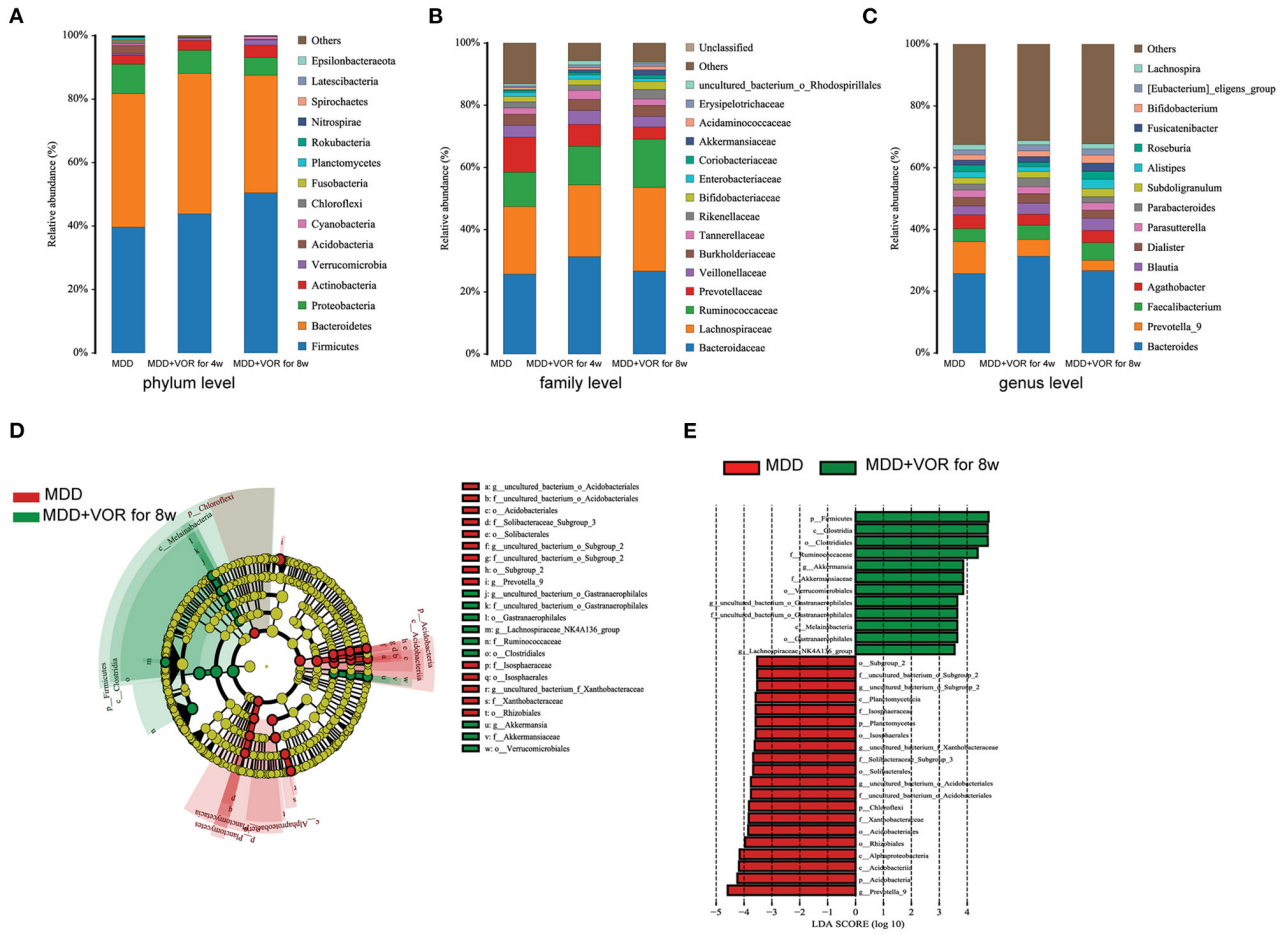
Taxonomic differences in the fecal microbiota between MDD patients before and after treatment. Comparison of relative abundance at the bacterial phylum **(A)**, family **(B)** and genus **(C)** levels before MDD treatment, MDD + VOR for 4 weeks, MDD + VOR for 8 weeks. LEfSe identified the most differentially abundant taxa between MDD + VOR after 8 weeks and before MDD treatment. The brightness of each dot is proportional to its effect size **(D)**. Taxa enriched before MDD treatment are indicated with a negative logarithmic discriminant analysis (LDA) score (red), and taxa enriched after 8 weeks of treatment (MDD + VOR for 8 weeks) have a positive score (green). Only taxa meeting an LDA significance threshold > 3.5 are shown **(E)**.

The degree of bacterial taxonomic similarity between metagenomics samples at the phylum, family and genus levels were analyzed to assess the composition of bacterial communities in the different groups. At the phylum level, Actinobacteria, Acidobacteria, Proteobacteria, and Bacteroidetes were significantly different before and after MDD treatment. With prolonged treatment time, Firmicutes and Actinobacteria significantly increased, while the proportions of Acidobacteria, Bacteroidetes, and Proteobacteria gradually decreased (Figure 5A). At the family level, with prolonged treatment time and improved depressive symptoms, Lachnospiraceae, Ruminococcaceae, Bifidobacteriaceae, Coriobacteriaceae, Akkermansiaceae, Acidaminococcaceae, and Erysipelotrichaceae increased, while the proportion of Prevotellaceae decreased (Figure 5B). At the genus level, the proportions of *Parasutterella, Prevotella-9, Dialister*, and *Agathobacter* gradually decreased with the relief of depressive symptoms, while the proportions of *Bacteroides, Faecalibacterium, Roseburia, Fusicatenibacter*, and *Bifidobacterium* gradually increased (Figure 5C).

Similarly, we used LEfSe analysis to estimate the impact of the abundance of each species on the differential effect and to identify the communities or species that significantly affected the sample partition. Because the abundance of the flora species in the MDD patients after 4 weeks of vortioxetine hydrobromide treatment was not significantly different from that before treatment and 8 weeks after treatment, we compared the key species that were significantly different before and after 8 weeks of treatment. At the phylum level, Proteobacteria and Acidobacteria were the most abundant before MDD treatment, and Firmicutes was the most abundant 8 weeks after vortioxetine hydrobromide treatment. At the genus level, *Prevotella-9* was the most abundant before MDD treatment, and *Akkermansia* was the most abundant 8 weeks after vortioxetine hydrobromide treatment (Figures 5D,E).

### Correlation Between Fecal Microbiota and HAMD Score

**Figure 6:**
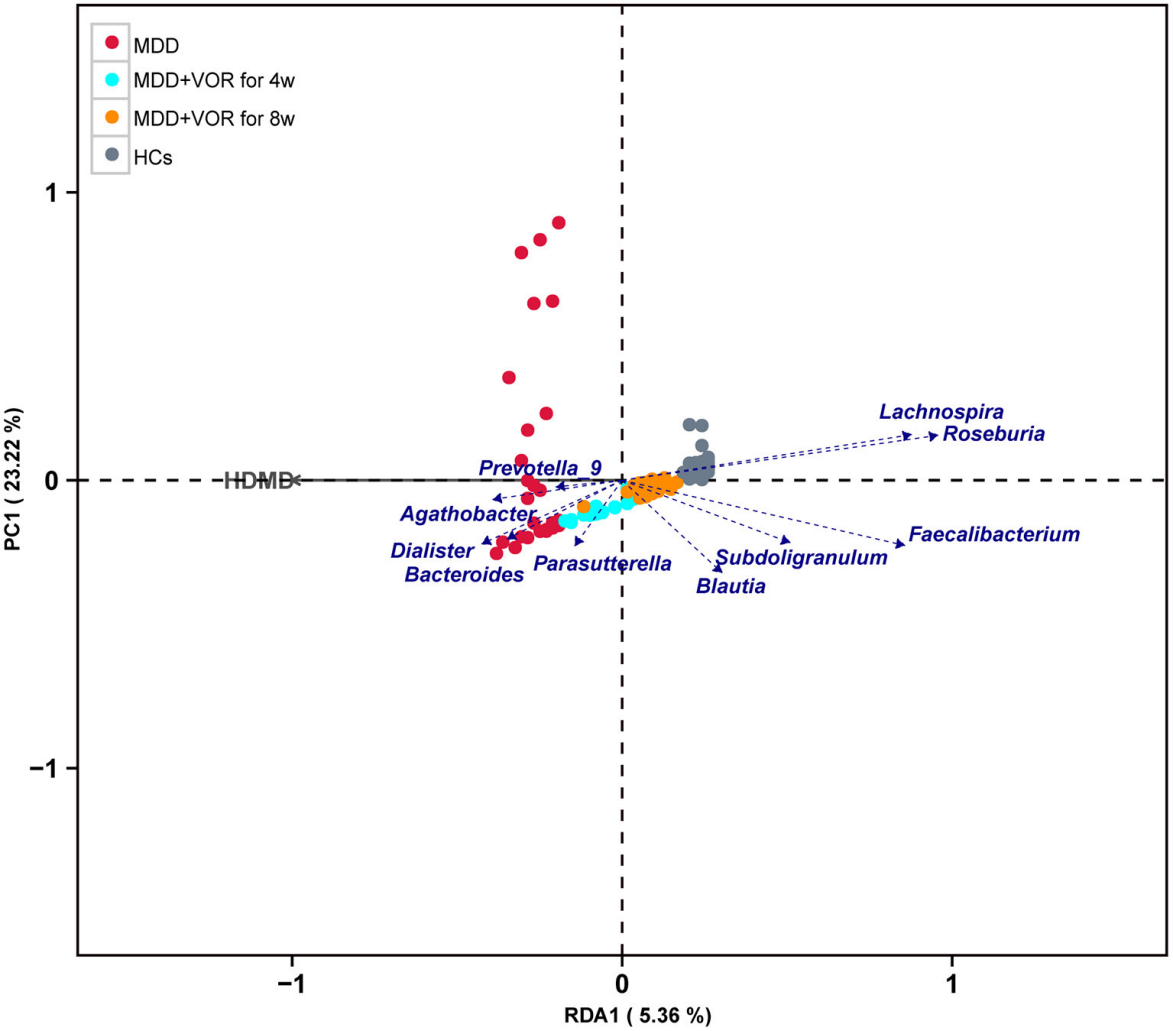
Correlations between fecal microbiota and HAMD score.

To further prove whether the MDD-associated clinical parameter directly contributes to alterations of the gut microbiota, we performed redundancy analysis (RDA) to link the HAMD score with the relative abundance of gut microbiota at the genus level. The results showed that *Lachnospira, Roseburia, Subdoligranulum, Faecalibacterium*, and *Blautia* were negatively correlated with the HAMD score, whereas *Parasutterella, Dialister, Bacteroides, Prevotella-9*, and *Agathobacter* were positively correlated with the HAMD score (Figure 6).

## Discussion

MDD is a common mental disorder that seriously affects the physical and mental health of patients and reduces their quality of life (19, 20). Previous studies on MDD have focused on genes, behaviors and neurotransmitters, but increasing attention has been paid to the role of environmental factors in the pathogenesis of MDD (21, 22). It has been confirmed that the gut microbiota is associated with depressive symptoms, including susceptibility to diseases and responses to drugs (23–25). Therefore, we hypothesized that certain specific gut microbiota in the intestinal tract may be associated with the efficacy of antidepressants; when patient symptoms improve, imbalanced gut microflora will undergo certain reconstruction. In this study, there were significant differences in the gut microbiota between MDD patients and HCs; short-term treatment with vortioxetine hydrobromide could reduce HAMD score, improve gut microbiota imbalance, and relieve the patient's degree of depression.

High-throughput sequencing analysis showed that the diversity and abundance of gut microflora species in MDD patients were higher than in HCs, a result that is consistent with the findings by Jiang et al. (26), albeit contrary to Naseribafrouei's study. Naseribafrouei et al. observed no significant differences in the structure or diversity of the gut microbiota in patients with MDD compared with HC participants (27). The inclusion criteria for subjects in our study were essentially the same as those in the study by Jiang et al., which might explain the consistent results. Our control group comprised healthy individuals and excluded individuals with psychological illness and mental illness. However, Naseribafrouei's study enrolled outpatients in the control group, and similar psychological pressure may exist among these patients. In addition, the subjects in the Naseribafrouei's study were older, and some patients took antihypertensive drugs. These differences may explain the inconsistent results between our study and Naseribafrouei's study. We generally believe that increased gut microflora diversity is conducive to human health, and it has been shown that a reduction in gut microbiota diversity is common in patients with obesity and inflammatory bowel disease and in those who take antibiotics (28, 29). Currently, the specific roles of gut microbiota diversity in MDD patients require further study.

In this study, we further compared the gut microbiota composition in MDD patients and HCs and found significant differences in the abundance distribution of many microbiota at the phylum and genus levels. The results showed that the proportions of Bacteroidetes and Proteobacteria was significantly higher and that of Firmicutes was significantly lower in MDD patients than in HCs, which is consistent with Jiang's study (26). However, other studies have shown that patients with MDD have a rich abundance of intestinal Firmicutes and Actinobacteria but lack Bacteroidetes and Proteobacteria (30). The difference between our results and the results of that study might be due to the slightly higher BMI of HCs in our study. In human and animal studies, a reduction in the abundance of Bacteroidetes has been associated with obesity (31). In addition, at the genus level, our study found that MDD patients had a significantly higher proportion of *Alistipes* and a lower proportion of *Faecalibacterium* and *Roseburia* than HCs. Studies have shown that *Alistipes* is associated with inflammation; therefore, *Alistipes* may cause depression through a variety of inflammatory pathways (32). As an indole-positive strain, *Alistipes* can affect TRP metabolism and may also interfere with 5-HT metabolism in the intestine (27). Other studies have also found that *Alistipes* is elevated in chronic fatigue syndrome and IBS; notably, patients with chronic fatigue syndrome often present depressive symptoms (33, 34). *Faecalibacterium* and *Roseburia* have been demonstrated by many studies to be negatively correlated with the severity of depression. Depression is associated with a mild chronic inflammatory response. *Faecalibacterium* is regarded to have a strong anti-inflammatory effect in gut microflora and therefore may be beneficial to host health (35). *Roseburia* and *Faecalibacterium* are butyric acid-producing bacteria; butyrate is one of the main products of the gut microbiota and can affect hippocampal function (36). The above results confirmed the presence of different flora in the intestinal tract of MDD patients. However, it remains to be seen whether the differential gut microbiota can be rebuilt with disease relief.

Therefore, in this study, we investigated the composition of gut microbiota in MDD patients treated with vortioxetine hydrobromide. Vortioxetine hydrobromide is an innovative drug for the treatment of adult depression. The discovery of this class of drugs has opened up a new era of multidimensional and multimodal mechanisms for the treatment of depression (37). The results of existing clinical studies have shown that vortioxetine hydrobromide helps improve the affective symptoms of depression and improves their cognitive symptoms; this improvement is independent of improvements in affective symptoms. This drug has been shown to facilitate comprehensive functional recovery and provides more choices and hope for patients with depression. In this study, the patients were administered vortioxetine hydrobromide for 4 and 8 weeks, and HAMD scores significantly decreased after the drug intervention. Our experimental results further confirmed the therapeutic effect of vortioxetine hydrobromide on MDD. We found abundance distribution differences in gut microbiota at both the phylum and genus levels. Compared with MDD patients before treatment, along with the alleviation of depressive symptoms, the proportions and amount of Bacteroidetes and Proteobacteria gradually decreased at the phylum level 4 and 8 weeks after treatment with vortioxetine hydrobromide, while the proportion and amount of Firmicutes gradually increased at the phylum level. With the alleviation of depressive symptoms, the proportions and amounts of these three types of bacteria in patients with MDD gradually approached the proportions and amounts of those in HCs. Therefore, treatment with vortioxetine hydrobromide may play a role in reconstruction of the gut microbiota in MDD patients. At the genus level, we found that the proportion and amount of *Bifidobacterium* significantly increased with treatment. *Bifidobacterium* is considered to have a beneficial effect on stress responses and depression (38). For example, the consumption of probiotic preparations (*Bifidobacterium longum* R0175) is negatively correlated with the HPA axis response and attenuates chronic stress-induced abnormal brain plasticity, neurogenesis reductions, and HPA axis hyperactivity in a depression mouse model (39). MDD patients often present with IBS, which is a typical psychological distress phenotype in the gastrointestinal system, and *Bifidobacterium* has a positive impact on IBS (40). Similarly, we also found that the amounts and proportions of *Faecalibacterium* and *Roseburia* significantly increased after 4 and 8 weeks of treatment and that *Faecalibacterium* was negatively correlated with the severity of depression. These results indicate that *Faecalibacterium, Roseburia*, and *Bifidobacterium* play important roles in the pathogenesis of MDD and the treatment response in patients with MDD and can even be used as important species for an effective response to depression treatment. The proportion and bacterial population of *Alistipes* in patients treated with vortioxetine hydrobromide decreased to some extent, but the decrease was not as significant as that for *Prevotella*. The abundance of *Alistipes*, an inflammatory bacterium in the gut, decreased after 4 weeks of treatment when MDD patients exhibited an antidepressant response. However, there was a certain increase at 8 weeks. It has been reported that the abundance of *Alistipes* is usually high in individuals who eat a simple diet (41), indicating that vortioxetine hydrobromide does affect the content of *Alistipes* in the gut microbiota. However, the dietary habits of 26 MDD patients did not change significantly over the short term in this study, suggesting that dietary intervention may play a role in depression. Finally, we found that the proportion of *Prevotella* in the gut microbiota of MDD patients after vortioxetine hydrobromide treatment significantly decreased. Lin et al. showed that a high abundance of *Prevotella* was associated with MDD patients, and changes in the proportions of *Prevotella* and *Klebsiella* were consistent with the HAMD scores (42). The above results indicate that reconstruction of microbial community structures in MDD patients treated with vortioxetine hydrobromide is more likely to be manifested by changes in the proportion of different microflora rather than changes in the abundance of different microflora. Therefore, changes in the proportion of specific genera in stool specimens will play an important role in the diagnosis and monitoring of MDD in the future. However, the conclusions are limited by the small sample size and the influence of potential uncontrollable factors on fecal microorganisms.

## Conclusions

The results from this study demonstrated that MDD is closely related to the gut microbiota. Vortioxetine hydrobromide may ameliorate depressive symptoms by promoting reconstruction of the gut microbiota. *Faecalibacterium, Roseburia*, and *Bifidobacterium* may be used as important species for an effective response to depression treatment. The results from this study provide a new perspective for the potential connection between gut microbiota and MDD and lay the foundation for the application of microecological-related therapeutic methods in the prevention and treatment of MDD. Future studies need larger sample sizes, increased long-term observation after antidepressant treatment, and verification through relevant animal experiments.

## Data Availability Statement

The datasets presented in this study can be found in online repositories. The names of the repository/repositories and accession number(s) can be found below: NCBI SRA; PRJNA685916.

## Ethics Statement

The studies involving human participants were reviewed and approved by Ethics Committee of China Medical University (Liaoning, China). The patients/participants provided their written informed consent to participate in this study. Written informed consent was obtained from the individual(s) for the publication of any potentially identifiable images or data included in this article.

## Author Contributions

JW and YT: conceptualization and writing-review and editing. XY and DoW: methodology. HZ: software and investigation. DaW, JL, and DoW: validation. XY: formal analysis, writing-original draft preparation, and visualization. DaW: resources. DoW and XY: data curation. DoW: supervision. YT: project administration and funding acquisition. All authors have read and agreed to the published version of the manuscript.

## Conflict of Interest

The authors declare that the research was conducted in the absence of any commercial or financial relationships that could be construed as a potential conflict of interest.
